# Peroxidase-Catalyzed and Photo-Oxidation of Tryptophan Results in Distinct Isomeric Tryptophan Dimers

**DOI:** 10.1021/acsomega.5c07535

**Published:** 2025-10-09

**Authors:** Marcela Morales, Daniel Villegas, Angélica Fierro, Mario Aranda, Maria Fernanda Hornos Carneiro, Michael J. Davies, Camilo López-Alarcón

**Affiliations:** † 28033 Pontificia Universidad Católica de Chile, Departamento de Química Física, Facultad de Química y de Farmacia, 7820436 Santiago, Chile; ‡ 42677 Comisión Chilena de Energía Nuclear (CCHEN), Centro de Tecnologías Nucleares para Ecosistemas Vulnerables, 7600713 Santiago, Chile; § Pontificia Universidad Católica de Chile, Departamento de Química Orgánica, Facultad de Química y de Farmacia, 7820436 Santiago, Chile; ∥ Pontificia Universidad Católica de Chile, Departamento de Farmacia, Facultad de Química y de Farmacia, 7820436 Santiago, Chile; ⊥ Universidade Estadual Paulista, Instituto de Biociências, Botucatu, SP 18618-689, Brazil; # University of Copenhagen, Department of Biomedical Sciences, Panum Institute, DK-2200 Copenhagen, Denmark

## Abstract

Heme peroxidases, including horseradish peroxidase (HRP), catalyze the oxidation of a wide variety of substrates by hydrogen peroxide (H_2_O_2_) via the peroxidase cycle of these enzymes. Oxidation of free tryptophan (Trp) by HRP/H_2_O_2_ has been previously reported, but the formation of tryptophan dimers (di-Trp), which are biologically relevant, has not been studied. Here, we report on di-Trp production arising from oxidation of free Trp, at pH 5.5 and 9.2, by HRP/H_2_O_2_, as determined by liquid chromatography–mass spectrometry (LC-MS/MS) and selected reaction monitoring (SRM). These data were compared with those from riboflavin-sensitized photo-oxidation, and the products were rationalized by *in silico* studies. Incubation of varying concentrations of Trp and H_2_O_2_ with HRP, irrespective of the pH, resulted in the consumption of ∼2 mol of Trp per mole H_2_O_2_. Formation of multiple di-Trp isomers was detected, using *m*/*z* 407 → 203 and *m*/*z* 407 → 390 transitions, with greater yields detected at pH 9.2 than 5.5. These results contrast with riboflavin-mediated photo-oxidation where one di-Trp dimer predominated as detected by the *m*/*z* 407 → 203 transition. *In silico* docking studies suggest di-Trp formation within the catalytic pocket of HRP, and subsequent release is a probable mechanism, although other alternative scenarios are also possible.

## Graphical abstract

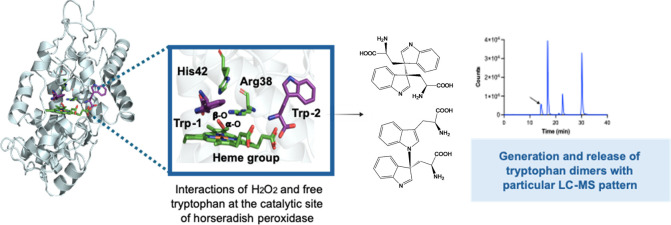

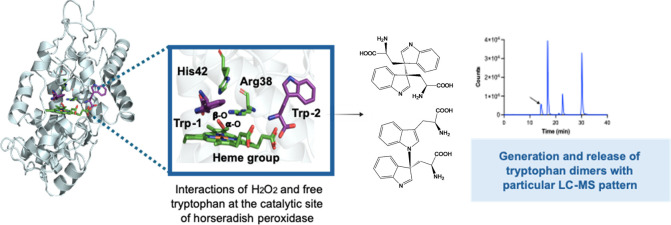

## 1 Introduction

Heme peroxidases, which are commonly found in plants, fungi, bacteria, and mammals, catalyze the oxidation of a variety of organic and inorganic substrates by hydrogen peroxide (H_2_O_2_).^1^ Some members of the superfamily exhibit multiple catalytic cycles (e.g., myelo-, lacto-, and eosinophil-peroxidases), whereas others such as the peroxidase from horseradish (horseradish peroxidase, HRP) have more limited enzymatic activity, with this limited to a peroxidatic cycle. As such, HRP is a useful model for enzyme-mediated peroxidase activity.^1,2^ The peroxidatic cycle involves three main steps and enzyme species, the resting state, and Compounds I and II. The first step involves two-electron reduction of H_2_O_2_ and concomitant oxidation of the resting Fe­(III) state heme to Compound I (a Fe­(IV)-oxyferryl species together with a porphyrin cation radical); this species is a powerful and efficient oxidant. In the second step, Compound I mediates one-electron oxidation of an electron-rich substrate, producing a substrate radical, a resting state porphyrin, and Compound II (a Fe­(IV)-oxyferryl species). The latter then reacts with a second substrate molecule, to give a second radical and the resting state of the enzyme. The crystal structure of HRP, the interactions of the heme group with substrates, and the catalytic mechanisms have been extensively studied.^1,3^ A histidine (His42) and an arginine (Arg38) residue, located on the distal side of the heme, are key residues for catalytic activity,^3^ with these participating in proton transfer reactions that facilitate Compound I formation and cleavage of the peroxide bond of H_2_O_2_ bound within the active site.^3^ Much of the data on the activity of HRP has been obtained from studies on phenols (e.g., cresol, chloro-phenols, and ferulic acid),^4–6^ dye degradation, and devices for H_2_O_2_ determination.^7–9^ Oxidation of the free amino acid tyrosine (Tyr) by HRP has been extensively studied (e.g., refs 10,11 ), with this resulting in the formation of the cross-linked species dityrosine (di-Tyr), from self-reaction of two tyrosyl radicals (TyrO^•^). This species is the major product under substrate and oxidant-limited conditions.^10–12^ Di-Tyr (and higher oligomers) is also formed by other peroxidases [e.g., myeloperoxidase (MPO) and lactoperoxidase],^13,14^ and associated with human diseases, where it has been used as a marker of radical formation and protein oxidation.^11,14–20^

Oxidation of indole compounds, such as free tryptophan (Trp), by HRP (and other peroxidases), in the presence of H_2_O_2_, has been examined, with one-electron oxidation to give tryptophanyl radicals (Trp^•^) identified as a key step,^21–24^ as would be expected from the similar reduction potentials of Tyr and Trp. The Trp reactions may be biologically relevant (e.g., in plasma^21^) as Trp-derived species play a key role in the control of vascular blood pressure and cardiovascular disease,^25,26^ and in cell signaling in some cancers.^27^ Studies on Trp oxidation by HRP/H_2_O_2_ have provided evidence for the formation of oxindolyl-alanine, dioxindolyl-alanine, as well as oligomers/polymers as products.^22^ Although di-Trp formation (from two Trp^•^) has been reported in biological systems (e.g., from processes involving light and radicals^28–32^ ), formation of di-Trp by HRP/H_2_O_2_ has not been investigated. Di-Trp has been implicated in the cross-linking of superoxide dismutase-1 by carbonate radicals from peroxidase-mediated bicarbonate oxidation,^31,32^ and involved in protein cross-linking in nuclear cataracts in human eye lenses.^28^ Di-Trp has also been detected on exposure of free Trp to γ-irradiation, carbonate radicals, UV-A light in the presence of the sensitizer kynurenic acid, and riboflavin-photosensitized reactions.^29,33–35^

In light of the biological relevance of di-Trp, and capacity of HRP/H_2_O_2_ to oxidize Trp,^22–24^ we have examined di-Trp formation by HRP/H_2_O_2_ with di-Trp dimers detected by liquid chromatography–mass spectrometry (LC-MS/MS). Multiple species have been detected and compared to those generated by riboflavin-mediated photosensitized reactions, and the products were rationalized by computational docking studies. These data demonstrate the complexity of the chemistry behind the production of these dimers and the pattern of the isomers formed by HRP.

## 2 Materials and Methods

### 2.1 Reagents

ABTS [2,2′-azinobis­(3-ethylbenzothiazoline-6-sulfonic acid)], horseradish peroxidase type VI (HRP), l-tryptophan (Trp, reagent grade, ≥98%), riboflavin, xylenol orange, formic acid, and methanol were purchased from Sigma-Aldrich. Hydrogen peroxide (H_2_O_2_) was supplied by Merck (Darmstadt, Germany). All solvents employed were HPLC grade.

### 2.2 Assessment of HRP Activity

1 mM ABTS^2–^ solutions were incubated at 25 °C in the presence of 2 nM HRP in 100 mM phosphate (pH 5.5, 6.2, 7.4) or 100 mM formate (pH 9.2) buffers in a quartz cuvette placed in a thermostated holder of an Agilent 8453 spectrophotometer. The reaction was initiated by the addition of H_2_O_2_ (100 μM final concentration) and monitored for 3 min. The concentration of the resulting ABTS radical cation (ABTS^•+^) was calculated from the absorbance intensity at 414 nm using a molar extinction coefficient of 31100 M^–1^cm^–1^.^36^ Enzyme activity was expressed as the concentration of ABTS^•+^ generated per min (μM min^–1^).

### 2.3 Oxidation of Trp by the HRP/H_2_O_2_ System

Typically, oxidation of free Trp by HRP/H_2_O_2_ was conducted by incubation of solutions containing 0.65 μM HRP, 500 μM H_2_O_2_, and 4 mM Trp in 100 mM phosphate (pH 5.5) or 100 mM formate (pH 9.2) buffers for 2 min at 25 °C. In some experiments, the H_2_O_2_ or Trp concentrations were varied between 0.25 and 1 mM, or 0.5–4 mM, respectively. After incubation, the HRP was removed by centrifugation using an Eppendorf 5415R centrifuge (14500 g, 10 min, 4 °C) employing 10 kDa Amicon Ultra filters. The resulting supernatants were collected for further analysis and chromatographic separation. In some experiments, the removed HRP (collected from the top section of the 10 kDa Amicon Ultra filters) was recovered and employed to induce oxidation of Trp. The magnetic circular dichroism (MCD) spectrum of this enzyme was determined at 1–2 μM (pH 9.2) and compared with that of the original enzyme (18 μM). MCD spectra were registered at 5 °C using a quartz cell with a 1 cm path length placed in the thermostated holder of a Jasco-1700 circular spectrometer equipped with an electromagnet (MCD581), set at a constant magnetic field of 15 kG.

### 2.4 Quantification of Trp Consumption

Trp consumption was quantified by using both its intrinsic fluorescence (FL) and also by LC-MS/MS. For FL measurements, aliquots of Trp/HRP/H_2_O_2_ solutions were taken, the HRP removed (see above), then diluted 400-fold in the respective buffer (100 mM phosphate pH 5.5, or 100 mM formate pH 9.2), and the corresponding FL emission spectra registered between 300 and 475 nm (with the excitation wavelength set to 290 nm) using a PerkinElmer LS-55 spectrofluorometer (Beaconsfield, U.K.). The fluorescence intensities at the emission maximum (360 or 365 nm for pH 5.5 and 9.2, respectively) were determined, and these values were compared to calibration curves (0–20 μM) generated using commercial samples of Trp, under the same experimental conditions.

Quantification of Trp consumption was also determined by LC-MS/MS, using an Ekspert system coupled to a triple quadrupole mass spectrometry detector (ABSciex 4500), with positive ionization (to form [M – H]^+^ ions) via electrospray ionization (ESI), as reported previously.^29^ Briefly, aliquots (10 μL) of purified (see above) Trp/HRP/H_2_O_2_ solutions (400-fold diluted with 0.1% formic acid) were injected on to a reversed phase column (μBondapak 150 × 3.9 mm^2^ RP-18 end-capped, particle size 10 μm; WAT086684, Waters) maintained at 30 °C, and eluted using a gradient of phase A (0.1% formic acid in water) and phase B (50:50 methanol: water containing 0.1% formic acid) at a flow rate of 0.8 mL min^–1^. Phase A was kept at 100% for the first 2 min, before decreasing to 20% over 30 min. Subsequently, phase A was returned to 100% over 35 min with a further equilibration phase of 5 min. Trp was detected by using a Selected Reaction Monitoring (SRM) mode in positive ion mode, employing the *m*/*z* 205 → 188 transition (corresponding to the loss of the α-ammonium group of Trp). The collision energy and declustering potentials were 17 and 9 V, respectively. A calibration curve was constructed using authentic commercial Trp under the same conditions (0–25 μM) and used to quantify Trp consumption.

### 2.5 Quantification of H_2_O_2_ Consumption

Consumption of H_2_O_2_ was determined by assessing the total hydroperoxide content of the samples by use of the ferric-xylenol orange (FOX) assay.^37^ Working solutions were generated by mixing reagent A (25 mM Mohr’s salt in 2.5 M H_2_SO_4_) with reagent B (125 μM xylenol orange with 100 mM sorbitol in water) at a 1:100 ratio. 200 μL of these solutions were placed in a well (96-well plate), aliquots (20 μL) of purified Trp/HRP/H_2_O_2_ samples were added, and the resulting mixtures were incubated at 25 °C for 20 min in the dark. The absorbance at 595 nm, corresponding to the xylenol orange-Fe^3+^ complex, was then measured using a Synergy HTX microplate reader (BioTek Instruments). The total peroxide concentration was determined using a calibration curve (0–90 μM) generated using commercial H_2_O_2_.

### 2.6 Chromatographic Separation and Detection of Di-Trp Dimers

Di-Trp dimers, generated in solutions of free Trp incubated with the HRP/H_2_O_2_ system (see Section 2.3), were separated by semipreparative liquid chromatography before analysis by LC-MS/MS. Purified Trp/HRP/H_2_O_2_ samples (see Section 2.3) were injected onto a semipreparative ZORBAX StableBond C18, nonend-capped (250 mm × 9.4 mm, 5 μm particle size) column placed in a chromatographic system consisting of a Jasco PU-4180 chromatograph, equipped with a diode-array detector. A gradient elution method was employed with a flow rate of 2.5 mL min^–1^, using 0.1% formic acid in water as phase A, and 0.1% formic acid in 50% methanol–water as phase B. The gradient consisted of 2 min of 100% phase A, followed by its linear decrease until 20% at 40 min. The proportion of phase A was then increased to 100% by 45 min, and kept at this level for 15 min. Fractions that eluted between 27 and 50 min were collected. These fractions were lyophilized (Martin Christ α 1–2 LDplus) and then redissolved in 0.1% formic acid (in water). Di-Trp dimers were analyzed and detected by LC-MS/MS using positive ionization (to form [M – H]^+^ ions) via electrospray ionization (ESI), using the same chromatographic conditions as those described in Section 2.4, changing the setup of the SRM mode for Q1 and Q3 to *m*/*z* = 407 → 203, and 407 → 390 transitions (Figure 1), and the collision energy and declustering potentials to 25 and 56 V, respectively.

**Figure 1:**
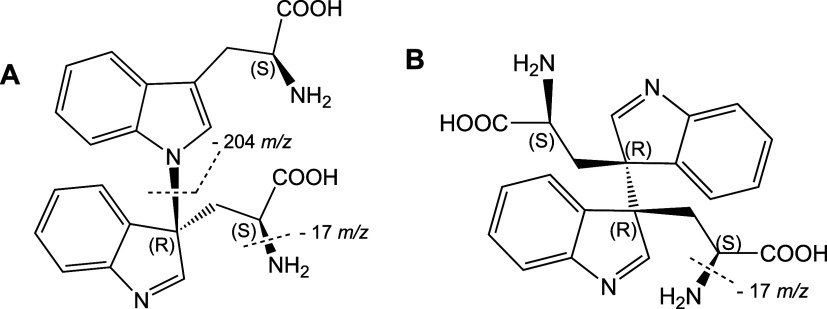
Proposed structures of the di-Trp species. Coupling of Trp^•^ leads to new nitrogen–carbon bonds (between N-1 and C-3 of the respective indole rings, panel A) or new carbon–carbon bonds (at C-3 of the respective indole rings, panel B). Structures indicate the stereochemistry used for docking studies, and the fragmentation involved in the *m*/*z* 407 → 203 and *m*/*z* 407 → 390 transitions used for LC-MS/MS analysis. It should be noted that additional structures of di-Trp species with the same fragmentation pattern are possible, with these arising from different diastereo- and regioisomers.

### 2.7 Formation and Analysis of Di-Trp Generated by Riboflavin-Photosensitized Reactions

Solutions of Trp (4 mM) were prepared at pH 9.2 in 100 mM formate buffer and illuminated, under aerobic conditions, for 6 min in the presence of 103 μM riboflavin using a 450 nm light emission diode (LED, 81.4 W/m^2^), placed in a Luzchem LED Illuminator (LEDi). After illumination, solutions were analyzed (without further purification) by LC-MS/MS as described in Section 2.6.

### 2.8 *In Silico* Studies

The crystal structure of HRP was obtained from Protein Data Bank (PDBid:1HCH) at a resolution of 1.57 Å.^38^ The HRP structure was submitted to the H++ server (http://newbiophysics.cs.vt.edu/H++/) to compute p*K*_a_ values of ionizable groups, and to add missing hydrogen atoms as required to model the reactions carried out at pH 5.5 and 9.2. The resulting HRP structure was used for docking studies of H_2_O_2_, Trp, and di-Trp dimers at pH values of 5.5 and 9.2. Di-Trp dimers corresponding to species generated by the formation of new covalent bonds between positions 3 and 1 of the respective indole rings on different Trp molecules (i.e., with a new carbon–nitrogen bond), and position 3 on both Trp molecules (i.e., with a new carbon–carbon bond) were examined (structures are displayed in Figure 1). Before docking studies, each ligand was energetically optimized, and the charge calculated using Spartan18 (version 1.3.0 Wavefunction, Inc., Irvine, CA, 2018) using the HF 6–31G* basis set at the Hartree–Fock level of theory. Then, complexes of HRP/H_2_O_2_, HRP/H_2_O_2_/Trp-1, HRP/H_2_O_2_/Trp-1/Trp-2, and HRP/H_2_O_2_/di-Trp were obtained using the AutoDock 4.0 suite,^39^ following a similar procedure to that described previously.^40^ First, docking analyses between HRP and H_2_O_2_ were developed, and the resulting complex (HRP/H_2_O_2_) was used for docking one Trp molecule (Trp-1). Docking of the second Trp molecule (Trp-2) with the HRP/H_2_O_2_/Trp-1 complex was then developed. To obtain the complexes, the volume was 60 × 60 × 60 for di-Trp dimers and 40 × 40 × 40 points for Trp and H_2_O_2_ with a grid-point spacing of 0.375 Å. The grid box of the complexes considered the heme group as center, and free Gibbs energies of these complexes were calculated using the AutoDock 4.0 suite and expressed as kcal mol^–1^. Each complex was selected considering energetic and probabilistic criteria, based on histograms showing the more probable conformations of the complexes, with 92% and 89% for the HRP-H_2_O_2_ complex, 41% and 49% for the HRP-H_2_O_2_-Trp-1 complex, and 60% and 69% for the HRP-H_2_O_2_-Trp-1-Trp-2 complex at pH 5.5 and 9.2, respectively.

### 2.9 Data and Statistical Analyses

Experimental data were obtained from at least three independent experiments, with each condition measured in triplicate. Data were processed using GraphPad software version 10.0 and expressed as the mean of the measurements. Statistical analyses were carried out using two-way ANOVA with Tukey′s post hoc tests, with *p* < 0.05 taken as statistically significant.

## 3 Results

### 3.1 Quantification of Trp Consumption by HRP-Catalyzed Reactions

The enzyme activity of HRP was determined by the one-electron oxidation of ABTS^2–^ leading to its stable radical, which has a well-characterized UV–visible spectrum.^38,41,42^ As presented in Figure S1, across the pH range studied, pH values of 5.5 and 9.2 showed the highest and lowest activity of HRP, respectively. Based on these results, and reported data showing a high efficiency of the enzyme to generate di-Tyr at alkaline pH,^6^ we selected pH 5.5 and 9.2 as conditions to assess the consumption of parent Trp, and formation of di-Trp, in solutions of free Trp, HRP, and H_2_O_2_. In order to minimize any potential secondary (over) oxidation of products, the experimental conditions employed an excess of Trp over the concentration of H_2_O_2_ and HRP. As presented in Figure 2A,B, incubation of solutions containing 4 mM Trp, 500 μM H_2_O_2_, and 0.65 μM HRP for 2 min at pH 9.2 resulted in an ∼10% decrease of the fluorescence intensity of Trp (maximum at 365 nm) as well as the area under the curve of its LC-MS/MS peak. Similar results were obtained at pH 5.5 (Figure S2), while incubation of Trp with H_2_O_2_ (500 μM), but without HRP (0.65 μM), or with HRP, but without H_2_O_2_ (control experiments), did not show changes in the FL intensity (Figure S3).

**Figure 2:**
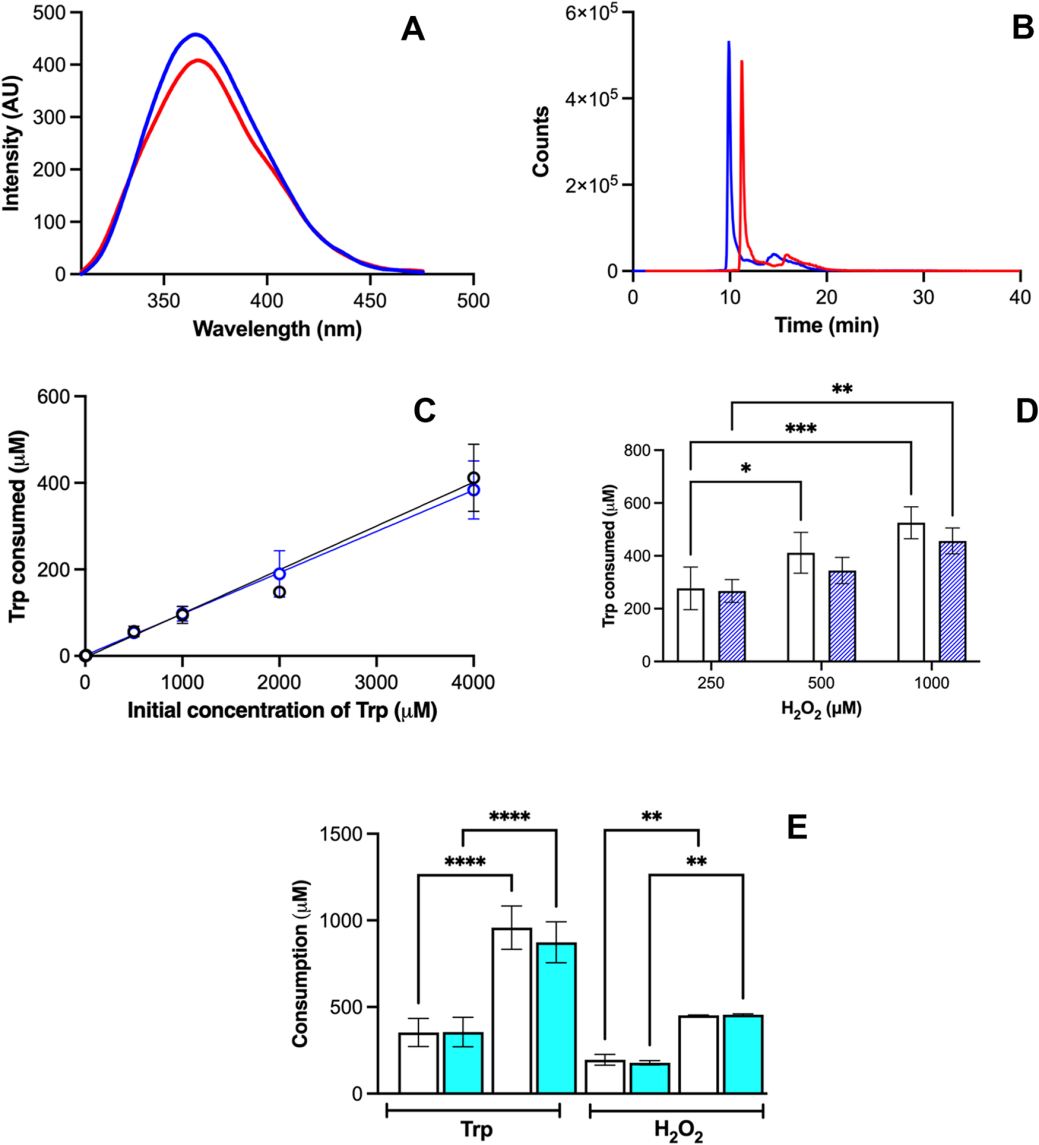
Incubation of HRP/H_2_O_2_ with Trp results in the consumption of Trp and H_2_O_2_. Panels (A) and (B) show the consumption of Trp (4 mM initial concentration) determined by its intrinsic fluorescence and by LC-MS/MS elicited by the 0.65 μM HRP/500 μM H_2_O_2_ system at pH 9.2. Blue and red lines show signals registered prior to and after 2 min incubation, respectively. For clarity, the red line in panel (B) is presented with an offset of 2 min with respect to the blue line. Panel C shows Trp consumption (0.65 μM HRP/500 μM H_2_O_2_) determined at different initial concentrations of Trp, while panel (D) shows Trp consumption (4 mM Trp, 0.65 μM HRP) determined at different H_2_O_2_ concentrations after 2 min incubation. White and blue open circles (panel C) or white and blue dashed bars (panel D) represent results obtained at pH 5.5 and 9.2, respectively. Panel (E) shows consumption of Trp and H_2_O_2_ from reaction (4 mM Trp, 0.65 μM HRP, 500 μM H_2_O_2_) carried out at pH 5.5 (white bars) and 9.2 (cyan bars) after 2 min (open bars) or 2 h (dashed bars) incubations. In panel (C), the data did not show any statistical differences, while in panels (D) and (E), the results showing statistical differences are indicated as follows: * *p* < 0.05, ** *p* < 0.01, *** *p* < 0.001, and **** *p* < 0.0001.

Solutions prepared with different Trp concentrations, incubated with fixed concentrations of H_2_O_2_ (500 μM) and HRP (0.65 μM), also showed a decrease of ∼10% in the fluorescence intensity, indicating a linear dependence of Trp consumption with its initial concentration (Figure 2C). Interestingly, the HRP recovered from the top section of the cutoff filters showed a similar activity at pH 9.2 as the original HRP solution, suggesting that the enzyme can be reused for new cycles of Trp oxidation. In a second cycle of reaction, using HRP recovered immediately after initial use, ∼415 μM Trp were consumed, while a slightly higher (but not statistically different) consumption was determined when recovered HRP was used 1.5 h after its removal (Figure S4). Additionally, the recovered enzyme showed comparable MCD and UV–vis spectra (Soret band at ∼400 nm, and MCD bands at ∼402 and ∼425 nm) to the ground state Fe–III species, as reported previously.^43^

In contrast to ABTS^2–^ oxidation (Figure S1), the consumption of Trp did not show a marked dependence on the reaction pH with similar consumption data detected at pH 5.5 and 9.2 (Figure 2C), which could be interpreted in terms of the participation of different ionizable groups in the interactions of ABTS^2–^ and HRP when compared to those involved in the interactions of Trp with the enzyme. Likewise, changes in the reaction pH did not result in any statistically significant differences in the extent of Trp consumption detected at different initial concentrations of H_2_O_2_. At both pH values, with initial H_2_O_2_ concentrations of 250, 500, and 1000 μM, consumption of Trp was ∼270, 400, and 520 μM, respectively, showing statistical differences between the data obtained at pH 5.5 at 250 and 500 μM, as well as at both pH values (5.5 and 9.2) between 250 and 1000 μM H_2_O_2_ (Figure 2D). As presented in Figure 2E, after 2 min of incubation of Trp/HRP/H_2_O_2_ mixtures, the extent of H_2_O_2_ consumption was ∼195 and ∼180 μM at pH 5.5 and 9.2, respectively, with these values being ∼50% of the Trp consumption (∼353 μM) at both pH values. Solutions incubated for 2 h showed higher consumptions of Trp and H_2_O_2_, with consumption of Trp of ∼960 and ∼880 μM at pH 5.5 and 9.2, and ∼450 μM H_2_O_2_ (Figure 2E).

### 3.2 Detection and Quantification of Di-Trp Formation by HRP-Catalyzed Reactions

Formation of di-Trp was detected by LC-MS/MS using SRM, with two transitions (*m*/*z* 407 → 203 and 407 → 390; see Figure 1). As presented in Figure 3A,B, the HRP/H_2_O_2_ system generated three major species as detected with the *m*/*z* 407 → 203 transition. An additional peak in the chromatograms (at ∼14 min, arrowed) corresponds to an artifact generated inside the LC-MS/MS detector, as corroborated by control samples containing only free Trp, which showed that the artifact peak and free Trp share the same elution time (Figure S5). As presented in Figure 3A,B, the intensities of the product signals depended on the reaction pH, with those arising from reactions at pH 9.2 significantly more pronounced than those from reactions at pH 5.5. Several peaks were also detected employing the transition *m*/*z* 407 → 390 (due to the loss of ammonia, as reported previously^35^), reflecting the presence of different species, with the signals from those detected at ∼20, ∼24, ∼26, and ∼28 min being of high intensity (Table 1). In a similar manner to the peaks detected with the *m*/*z* 407 → 203 transition, some of the signals detected using the *m*/*z* 407 → 390 transition showed higher intensities from reactions carried out at pH 9.2 compared to pH 5.5, with this being particularly noticeable for the peak detected at ∼24 min (Figure 3D versus E). These signals are ascribed to di-Trp species generated by the reactions elicited by the HRP/H_2_O_2_ system, and not to the fragmentation of oligomers (or other high molecular mass species) in the mass detector induced by the SRM mode, since the extracted ion chromatograms (EIC) using *m*/*z* = 407 showed a similar number of signals (data not shown).

**Figure 3:**
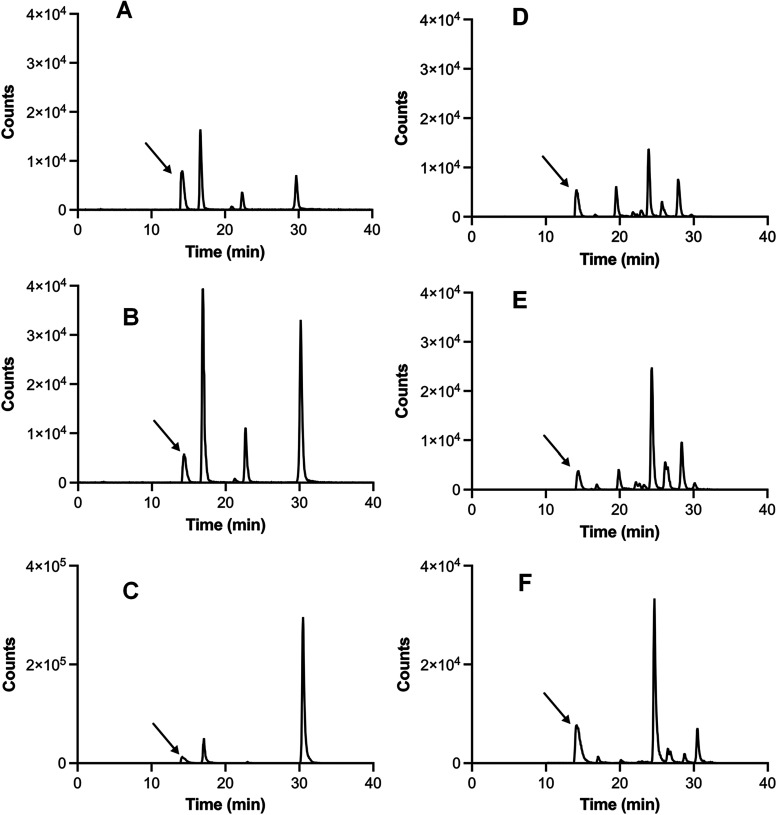
Production of di-Trp by the Trp/HRP/H_2_O_2_ system, and photochemical reactions mediated by riboflavin, determined by LC-MS/MS. Panels (A), (B), and (C) show extracted ion chromatograms for *m*/*z* 407 → 203 for Trp/HRP/H_2_O_2_ solutions incubated at pH 5.5 and 9.2 (panels A and B, respectively), and Trp-riboflavin solutions illuminated at pH 9.2 (panel C). Panels (D), (E), and (F) show extracted ion chromatograms for the *m*/*z* 407 → 390 transition determined for reactions induced by the HRP/H_2_O_2_ system, carried out at pH 5.5 and 9.2 (panels D, and E, respectively), and signals for the same transition for di-Trp generated by illumination of solutions containing riboflavin and Trp (panel F). The arrow present in each panel indicates an artifact signal detected in all experiments, including controls, with the same retention time as parent Trp.

**Table 1:** Retention Times and Intensities of LC-MS/MS Signals Detected for the Di-Trp Dimers Detected Using SRM and the Indicated *m*/*z* Transitions, for Samples Analyzed after 2 Min Reaction for HRP/H_2_O_2_/Trp Reaction Systems (0.65 μM HRP, 500 μM H_2_O_2_, 4 mM Trp)

	pH 5.5		pH 9.2	
transition (*m*/*z*)	retention time (min)	intensity (counts)	retention time (min)	intensity (counts)
407 → 390	16.7	3.8 × 10^2^	16.9	9.9 × 10^2^
	19.6	6.0 × 10^3^	19.9	3.9 × 10^3^
	21.8	8.7 × 10^2^	22.2	1.4 × 10^3^
	23.0	1.2 × 10^3^	23.3	9.2 × 10^2^
	24.0	1.4 × 10^4^	24.4	2.5 × 10^4^
	25.8	2.9 × 10^3^	26.2	5.4 × 10^3^
	27.9	7.5 × 10^3^	28.5	9.4 × 10^3^
	29.8	2.7 × 10^2^	30.2	6.9 × 10^2^
407 → 203	16.7	1.6 × 10^4^	16.9	3.9 × 10^4^
	22.4	3.5 × 10^3^	22.7	1.1 × 10^4^
	29.7	6.9 × 10^3^	30.2	3.3 × 10^4^

In order to assess whether these di-Trp products generated by HRP are common to other oxidation pathways, free Trp was subjected to riboflavin-mediated photo-oxidation. Illumination for 6 min (with a light emission diode that emits light at 450 nm) of solutions containing 4 mM Trp, and 103 μM riboflavin in 100 mM formate buffer pH 9.2, in the presence of O_2_, resulted in the detection by LC-MS/MS a *m*/*z* 407 → 203 transition for two peaks with retention times of ∼17 and ∼30 min (Figure 3C). The signal from the species registered at ∼30 min was significantly more intense than that from the species giving a peak at ∼17 min. As with the HRP/H_2_O_2_ system, further peaks were detected employing the *m*/*z* 407 → 390 transition (Figure 3F).

### 3.3 *In Silico* Analysis of the Interactions of Trp and Di-Trp with HRP

The above experimental data were rationalized by docking studies using the crystal structure of HRP reported in the Protein Data Bank (ID: 1HCH). After optimization of the structure, complexes between HRP and H_2_O_2_ at pH 5.5 and 9.2 were obtained with free Gibbs energies of −3.18 and −3.25 kcal mol^–1^, respectively. As expected, at both pH values, the H_2_O_2_ was observed to be located close to the iron atom of the heme group (Figures S6 and 4, for pH 5.5 and 9.2, respectively). These complexes (HRP/H_2_O_2_) were employed for docking analysis with free Trp; first, docking between HRP/H_2_O_2_ with one Trp molecule (Trp-1) was developed, showing that Trp-1 occupied the inner site of the enzyme with free Gibbs energies of −4.73 and −4.81 kcal mol^–1^, at pH 5.5 and 9.2, respectively. Docking between the HRP/H_2_O_2_/Trp-1 complex with a second Trp molecule (Trp-2) resulted in the complexes presented in Figures S6 and 4, where Trp-2 interacted with the HRP/H_2_O_2_/Trp-1 complex with free Gibbs energies of −3.16 and −3.07 kcal mol^–1^, at pH 5.5 and 9.2, respectively. As presented in Figures 4 and S6, the two Trp molecules studied were observed to be located close to the heme group, as well as His42 and Arg38, though the positions were dependent on the pH value modeled, with these differing significantly between the two pH values examined. The distances between these species are listed in Table 2. As can be seen from Figures 4 and S6, one of the Trp residues (Trp-1) is located close to the α-O, β-O, and Fe atoms, though in different orientations, with values of 6.6, 5.3, and 7.3 Å at pH 5.5, and 8.2, 7.0, and 8.7 Å at pH 9.2, respectively (Table 2). In contrast, Trp-2 showed much greater pH-dependent effects, with this being located on the opposite side of the structure (Figures 4 and S6) and longer distances than Trp-1 to the same atoms, with values varying between 13.0 and 15.3 Å. Both Trp molecules are located close to His42 and Arg38; however, Trp-1 showed shorter distances with 6.2 and 7.3 Å for His42, and 4.1 and 3.7 for Arg38, respectively. The proximity of Trp-1 to Arg38 is consistent with cation-π interactions. Both Trp molecules also showed close interactions with other residues and particularly Phe179, Phe143, and Asp150 (Table 2 and Figure S7). Docking analysis showed that the distances between the two Trp molecules (Trp-1 and Trp-2) are ∼12.0 and ∼13.1 Å at pH 5.5, and 9.2, respectively (Table 2, Figures 4 and S6).

**Figure 4:**
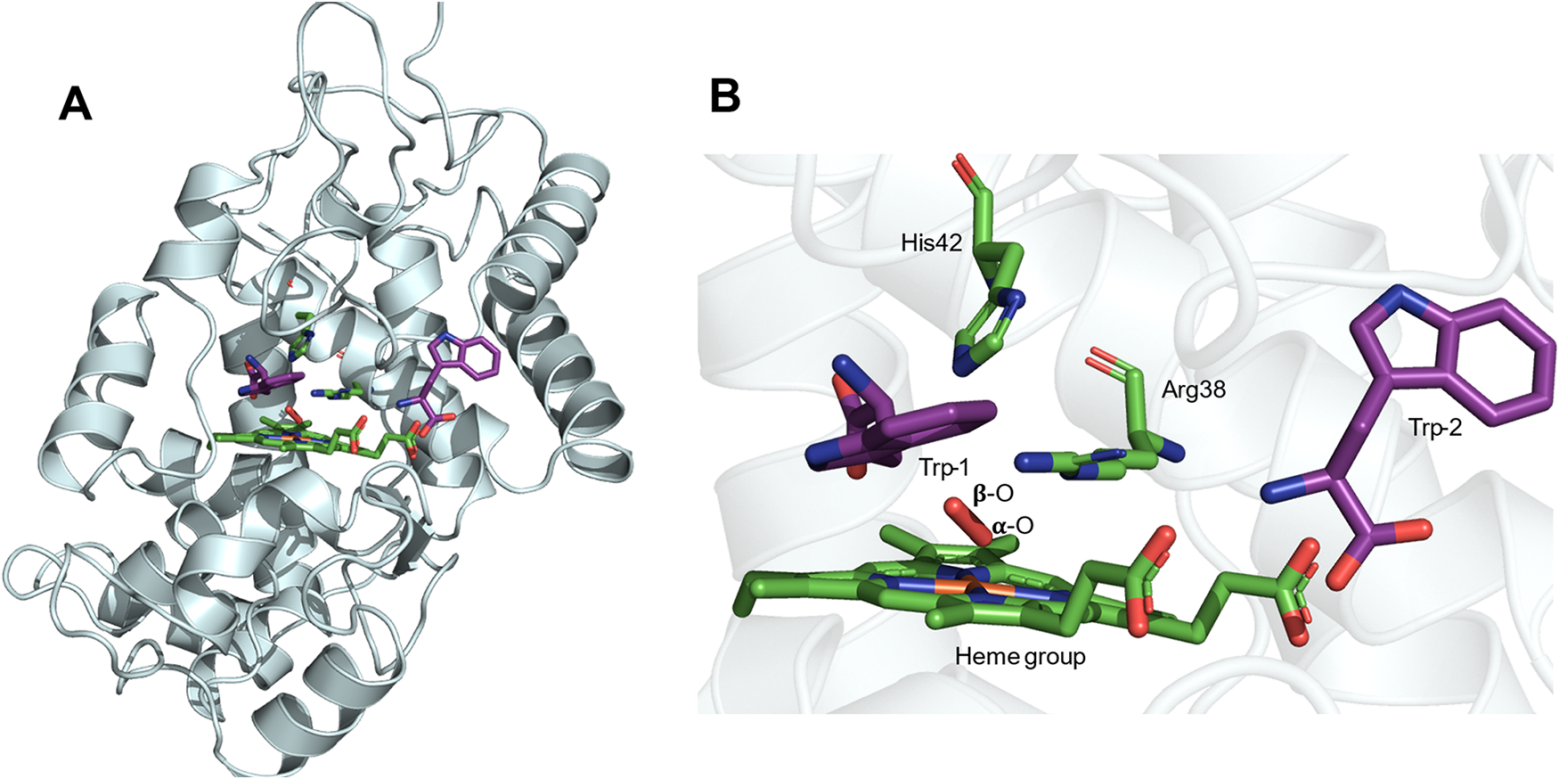
Docking analysis of the HRP/H_2_O_2_ complex in the presence of 2 molecules of Trp. Panels (A) and (B) depict the overview of the complex and an expanded region of the zone where the Trp residues interact with the heme group, His42 and Arg38. The complex was simulated at pH 9.2 using the crystal structure of the enzyme with PDB entry 1HCH. Docking analysis was carried out as described in the Section 2. The obtained complex (HRP/H_2_O_2_/Trp-1) showed that Trp-1 occupied the inner site of the enzyme. Docking between HRP/H_2_O_2_/Trp-1 with the second Trp molecule (Trp-2) resulted in the complex presented in this Figure.

**Table 2:** Distances between Indicated Atoms and Principal Interactions between Trp and HRP Determined by Docking Analysis of the HRP/H_2_O_2_/Trp System with a 1:2 Molar Ratio of HRP to Trp^a^

	distances to Trp-1 (Å)		distance to Trp-2 (Å)	
	pH 5.5	pH 9.2	pH 5.5	pH 9.2
α-O	6.6	8.2	14.8	13.2
β-O	5.3	7.0	14.2	13.0
Heme (Fe)	7.3	8.7	15.3	13.9
His42	6.2	7.3	13.0	12.6
Arg38	4.1 (cation-π)	3.7 (cation-π)	15.3	10.0
Trp-1	---	---	12.0	13.1
Phe179	5.6 (t-shape)	4.4 (t-shape)	4.4	11.9
Phe143	8.1	9.3	5.5 (stacking)	21.0
Asp150	16.6	18.5	6.3	30.8
Arg75	24.6	15.8	10.8	4.8 (cation-π)

a For simplicity, Trp molecules were denoted as Trp-1 and Trp-2, as indicated in Figure 4. The hydrogen atoms present on the β-O and α-O of H_2_O_2_ are located at 2.5 and 4.4 Å to His42 and Arg38, respectively, at pH 5.5 and 9.2. The distances were calculated from the center of the indole ring of Trp to the imidazole ring of His, a terminal nitrogen of the guanidinium group of Arg, the center of the phenyl group of Phe, and a terminal oxygen of the side chain of Asp.

Similar docking studies were attempted using di-Trp in place of the two free Trp residues. Both species with a covalent bond between position 3 and position 1 (i.e., a species containing a new carbon–nitrogen bond), and species with a new carbon–carbon bond between the two carbons at position 3 of the respective indole rings, were examined (for structures, see Figure 1). In neither case was an association detected with the HRP-H_2_O_2_ complex, with positive free Gibbs energies determined at both pH 5.5 and 9.2, consistent with endergonic associations in both cases. The C–C (position 3– position 3, Figure 1B) di-Trp species showed values of +4.2 and +39.3 kcal mol^–1^ at pH 5.5 and 9.2, respectively, while values for the di-Trp species with a new C–N bond (position 3– position 1, Figure 1A) yielded values of +16.8 and +26.7 kcal mol^–1^ at the same pH values.

## 4 Discussion

In agreement with the known high efficiency of HRP/H_2_O_2_ to induce ABTS^2–^ oxidation (Figure S1), our data show an efficient oxidation (as measured by consumption) of Trp mediated by this system. Under our experimental conditions, ∼400 μM Trp was consumed after 2 min incubations (probably forming oxygenated derivatives as principal products and to a more minor extent, di-Trp) as determined from the decrease of its intrinsic fluorescence and LC-MS/MS peaks (Figure 2). This high efficacy of HRP/H_2_O_2_ to induce Trp consumption is in contrast to that reported by Nguyen and co-workers,^22^ where changes in the UV–visible spectrum of Trp, induced by HRP/H_2_O_2_ were observed at long-time (hour) incubations, with this being faster at pH 4.29 than 7.0.^22^ Differences in the experimental conditions (Trp and H_2_O_2_ concentrations, enzyme activity, and pH), as well as interference from multiple species, including reaction products, absorbing at wavelengths similar to that of Trp, could explain these differences. Interestingly, Nguyen and co-workers,^22^ also presented evidence for the formation of oligomers after 10 days of incubation of free Trp with HRP/H_2_O_2_; however, they did not investigate the production of di-Trp. Oxidation of Trp is also catalyzed by MPO, an enzyme released by activated neutrophils at sites of inflammation in multiple pathologies.^44,45^ Trp is rapidly oxidized by Compound I of MPO, but reaction with (the less powerfully oxidizing) Compound II species is less rapid.^21^ Furthermore, in both the absence and presence of chloride (an alternative substrate for Compound I of MPO), Trp-mediated inactivation has been reported.^46^ Nonetheless, in the presence of species (e.g., superoxide radical anions) that are able to reduce Compound II back to the ground state, one-electron oxidation of Trp to produce Trp^•^ has been suggested as a significant reaction of MPO.^21^ The fate of MPO-generated Trp^•^ and the role of these radicals, and dimer formation, in human diseases, has not been explored yet, though other Trp-derived products play major roles in cell signaling and biological processes.^25–27^ In the current work, formation of di-Trp was detected by LC-MS/MS using SRM mode with *m*/*z* 407 → 203 and 407 → 390 transitions. These transitions correspond to the formation of fragment ions from di-Trp dimers (Figure 1) previously detected and characterized in studies of Trp oxidation mediated by riboflavin-photosensitized reactions, γ-irradiation, and carbonate radicals.^29,34,35^ In these systems, formation of di-Trp occurs via self-reactions of Trp^•^ generated by one-electron oxidation of free Trp.^29,34,35^ The *m*/*z* 407 → 203 transition, assigned to release of a Trp molecule from the dimer, has been associated with fragmentation of the C–N dimer (i.e., the species arising from coupling between radical centers located at N1 and C3 on the indole ring, Figure 1A). In contrast, the *m*/*z* 407 → 390 transition has been ascribed to the loss of ammonia from any of the dimers (Figure 1B). Fragmentation of the C3–C3 dimer, with loss of Trp, in a process analogous to that assigned to the C–N dimer (see above) does not appear to be facile as the C–C bond is stronger than the C–N bond, and therefore the C3–C3 adducts are more stable than the N1–C3 species.^29,34,35^ As presented in Figure 3 and Table 1, the retention times of the more intense peaks registered using both *m*/*z* transitions (∼17, ∼22, and ∼30 min, and ∼20, ∼24, ∼26, and ∼28 min for the *m*/*z* 407 → 203 and 407 → 390 transitions, respectively) showed that at least seven di-Trp dimers are present. Production of these species could be explained by the presence of both regioisomers (arising from spin delocalization across the indole ring) and also diastereomers. Despite the similar consumption of Trp at pH 5.5 and 9.2, the signals ascribed to di-Trp adducts showed LC-MS/MS intensities at pH 9.2 that were higher than those at pH 5.5. These results show that while the stoichiometry of Trp oxidation mediated by HRP/H_2_O_2_, which involve production of oxygenated products as principal products (Reactions 1–4), is not affected by pH, radical–radical reactions leading to di-Trp appear to be favored at basic pH (Reaction 5).

1 $$\mathrm{HRP}\left({\mathrm{Fe}}^{3+}\right)+H_{2}O_{2}\to c\mathrm{ompound I}+H_{2}O$$

2 $$c\mathrm{ompound I}+\mathrm{Trp}\to c\mathrm{ompound II}+{\mathrm{Trp}}^{•}$$

3 $$c\mathrm{ompound II}+\mathrm{Trp}\to \mathrm{HRP}\left({\mathrm{Fe}}^{3+}\right)+{\mathrm{Trp}}^{•}$$

4 $${\mathrm{Trp}}^{•}+O_{2}\to \mathrm{oxygenated products}$$

5 $${\mathrm{Trp}}^{•}+{\mathrm{Trp}}^{•}\to \mathrm{di}‐\mathrm{Trp}$$

Participation of TrpH^•+^ (p*K*_a_ 4.3,^47^) can be disregarded, since its neutral state would be the principal form present at the pH values studied. However, electrostatic (ionic) repulsions between the protonated α-amino group of Trp could lower the extent of di-Trp production at pH 5.5, as this amino group has a p*K*_a_ of 9.3. Thus, the higher fraction of the neutral α-amino group present at pH 9.2 probably favors radical–radical coupling. This agrees with the suggested effect of the charge on dimerization of free Trp, as well as the total charge of Trp-containing peptides on the second order rate constants (*k*_2_) of Reaction 5, where higher *k*_2_ values have been reported for *N*-acetyl-Trp (*k*_2_ 6.1 ± 0.1 x 10^8^ M^–1^ s^–1^) and *N*-acetyl-Trp-O-Me (*k*_2_ 6.4 ± 0.2 x 10^8^ M^–1^ s^–1^) than free Trp (*k*_2_ 5.0 ± 0.1 x 10^8^ M^–1^ s^–1^).^35^

It should be noted that this analysis only considers the effect of pH on the reactions of Trp or Trp^•^, and excludes any interactions between the binding/catalytic site of HRP and Trp or Trp^•^. In this context, reports on the formation di-Tyr by HRP/H_2_O_2_/Tyr suggest that the protonated form of the amine group of Tyr (p*K*_a_ 9.1) can participate in the HRP catalytic cycle at basic pH, allowing protonation of Compound I, probably explaining the higher extent of di-Tyr production at basic pH.^6^ Based on these data as well as that reported here, it appears that the effect of pH on di-Trp formation involves multiple factors. While consumption of Trp was not sensitive to pH changes, a higher extent of di-Trp formation was detected at pH 9.2. Additionally, the pattern of di-Trp species detected with HRP/H_2_O_2_ appears to differ from that reported previously,^34^ and detected here on illumination, using a 450 nm LED, of free Trp in the presence of riboflavin at pH 9.2. As previously reported,^34^ photosensitized reactions by riboflavin generate Trp^•^ by electron transfer between the excited triplet state of riboflavin and free Trp (type 1 mechanism). In this system, only two di-Trp species (eluting at ∼17 and ∼30 min in LC-MS/MS chromatograms) were detected when the *m*/*z* 407 → 203 transition was used, while multiple peaks were detected employing the *m*/*z* 407 → 390 transition (Figure 4). The data from the *m*/*z* 407 → 203 transition are similar to that reported for di-Trp induced by carbonate radicals.^29^ Interestingly, a comparison of signals detected with the *m*/*z* 407 → 203 transition in the presence of riboflavin, with those for the HRP/H_2_O_2_ system, shows that the peaks at ∼17 and ∼30 min are common. However, differences were also observed. The HRP/H_2_O_2_ system gave rise to additional signal at ∼23 min, in addition to those at ∼17 and ∼30 min, with these species detected in a 3.5:1.0:3.0 ratio, while the two species detected in the riboflavin-mediated photoreactions at ∼17 and ∼30 min were detected at a ratio of 1.0:7.0. These results show that the HRP system generates both additional species and an altered ratio of products to those mediated by riboflavin/light, and carbonate radicals.^29^ These additional signals may reflect a more diverse formation of di-Trp dimers when compared to the riboflavin system (Figure 3). The *in silico* analysis of the HRP/H_2_O_2_ complex and its interactions with Trp and di-Trp (Figures 4 and S6) provides potential explanations for these differences. Two scenarios can be envisaged:

1.- Formation of Trp^•^ inside the catalytic pocket of HRP followed by their release, and subsequent self-reaction of two Trp^•^ in bulk solution to give di-Trp.
2.- Formation and self-reactions of Trp^•^ inside the catalytic pocket of HRP, followed by release of di-Trp dimers.

As the LC-MS/MS results show a different pattern of di-Trp species with the HRP/H_2_O_2_ system when compared to the riboflavin-mediated photo-oxidation, the first scenario appears less probable. For the second scenario, two potential factors may be of importance in favoring the generation of additional/alternative dimers. One of these involves electronic interactions between Trp^•^ and the protein with subsequent perturbation of the spin density at the individual atoms of the indole such that dimerization becomes more favored at alternative sites to that observed in free solution. The second possibility involves interactions between Trp^•^ and the protein to make reactions that are kinetically slower (but thermodynamically favorable) than those observed in free solution more likely. This may involve a decrease in the rate constant for the dimerization from that observed in free solution (*k*_2_ 4.6–7.3 × 10^8^ M^–1^ s^–1^ at pH 10,^48^ and 5.0 × 10^8^ M^–1^ s^–1^ at pH 7.4 ^35^) or an enhancement in the rate of slower alternative reactions. The current data does not allow these possibilities to be distinguished, and the reported kinetic data may be composites for dimerization via alternative sites, as multiple species have been detected in product studies.^33,35^ In either case, it appears that once dimerization has occurred, di-Trp is released rapidly from the enzyme pocket as the docking studies indicate that the interactions of di-Trp with HRP are endergonic. The formation of two Trp^•^ within the catalytic pocket of HRP may arise via rapid electron transfer between Trp-1 and Trp-2, with Trp-1 being oxidized initially by Compound I (to give Trp-1^•^), but with this subsequently repaired by electron transfer from Trp-2 (to give Trp-2^•^ and a repaired Trp-1). Trp-1 may then be oxidized by Compound II, to give a pair of Trp^•^ bonds within the active site that then combine to give di-Trp. Alternative scenarios are also possible, including interactions of Trp^•^ with the surface of HRP, followed by the reaction of two Trp^•^ to generate di-Trp.

Whether other peroxidases (e.g., MPO, lactoperoxidase, and cytochrome c peroxidase)^12,21,44,45,49^ also generate altered ratios of dimers is unknown and remains to be explored. Similarly, it would be of interest to examine whether different isomer patterns are generated on oxidation of Tyr to di-Tyr (where both carbon–carbon and carbon–oxygen linkages can be generated, with the former predominating)^29,50,51^ when these species are formed enzymatically or in free solution. Although the current data show effective formation of di-Trp species by HRP/H_2_O_2_, the efficacy of this process appears to be lower than that reported for the formation of di-Tyr from the oxidation of Tyr induced by the same enzyme system.^52^ We estimate that ∼3.0 μM di-Trp (expressed as Trp equivalents) is produced, representing ∼1% of the total consumption of Trp. Considering H_2_O_2_ as the limiting factor, this value would indicate that ∼0.006 mol of di-Trp were produced by each mole of H_2_O_2_. Similar analysis for the data reported by Giulivi and Davies^52^ showed that ∼0.0125 mol of di-Tyr were produced by per mole of H_2_O_2_, suggesting that di-Trp species were produced at a 2-fold lower ratio. Nonetheless, for a suitable comparison of the efficiency of the HRP/H_2_O_2_ system to generate either di-Trp or di-Tyr from the respective one-electron oxidation of the parent amino acids, new investigations using the same experimental conditions are necessary.

## 5 Conclusions

The data from this study indicate that production of di-Trp mediated by HRP and H_2_O_2_ is an efficient process and results in a specific pattern of dimers (detected by LC-MS/MS), which can be explained by specific interactions between Trp and the HRP-H_2_O_2_ complex. These reactions may facilitate the synthesis of specific di-Trp species for future investigations in which large amounts of di-Trp species are required. This work also provides important information on the mechanisms of generation of di-Trp, which is relevant for a better understanding of their formation in biological systems and their association with the onset and development of human pathologies where these species arise via peroxidase enzymes or direct oxidation processes.

## Supplementary Material



## Notes

Enzymatic activity of HRP determined by oxidation of ABTS^2–^ to its cation radical (Figure S1); consumption of Trp induced by the HRP/H_2_O_2_ system at pH 5.5 (Figure S2); fluorescence spectra of Trp corresponding to controls of the reactions, Trp solutions incubated in the absence or presence of HRP and H_2_O_2_ (Figure S3); activity of recovered HRP determined by Trp consumption and MCD (Figure S4); analysis by LC-MS/MS of Trp solutions (in the absence or presence of HRP and H_2_O_2_) detected with transitions corresponding to di-Trp (Figure S5); docking analysis of the Trp/HRP/H_2_O_2_ complex at pH 5.5 (Figure S6); and main interactions of Trp with residues of HRP in the Trp/HRP/H_2_O_2_ complex (Figure S7) (PDF)
