# Supplementary material for: Peroxidase-Catalyzed and Photo-Oxidation of Tryptophan Results in Distinct Isomeric Tryptophan Dimers
Source: ACS Omega. 2025 Oct 9;10(41):48991–9001. doi: 10.1021/acsomega.5c07535 (PMC12547766; doi:10.1021/acsomega.5c07535)
Supplement: Supplementary file 1 [file ao5c07535_si_001.pdf]

Peroxidase-catalyzed- and photo- oxidation of tryptophan results in distinct isomeric tryptophan dimers

Supporting Information

Marcela Morales,[a] Daniel Villegas,[b] Angélica Fierro,[c] Mario Aranda,[d] Maria Fernanda Hornos Carneiro,[d,e] Michael J. Davies,[f] Camilo López-Alarcón[a*]

[a] Pontificia Universidad Católica de Chile, Departamento de Química Física, Facultad de Química y de Farmacia, Chile.
[b] Comisión Chilena de Energía Nuclear (CCHEN), Centro de Tecnologías Nucleares para Ecosistemas Vulnerables, Chile.
[c] Pontificia Universidad Católica de Chile, Departamento de Química Orgánica, Facultad de Química y de Farmacia, Chile.
[d] Pontificia Universidad Católica de Chile, Departamento de Farmacia, Facultad de Química y de Farmacia, Chile.
[e] Universidade Estadual Paulista, Instituto de Biociências, Brazil.
[f] University of Copenhagen, Department of Biomedical Sciences, Panum Institute, Denmark.

Author to whom correspondence should be addressed: C. López-Alarcón; e-mail: clopezr@uc.cl

**Supplementary Figures**	**Page**
Figure S1	3
Figure S2	4
Figure S3	5
Figure S4	6
Figure S5	7
Figure S6	8
Figure S7	9

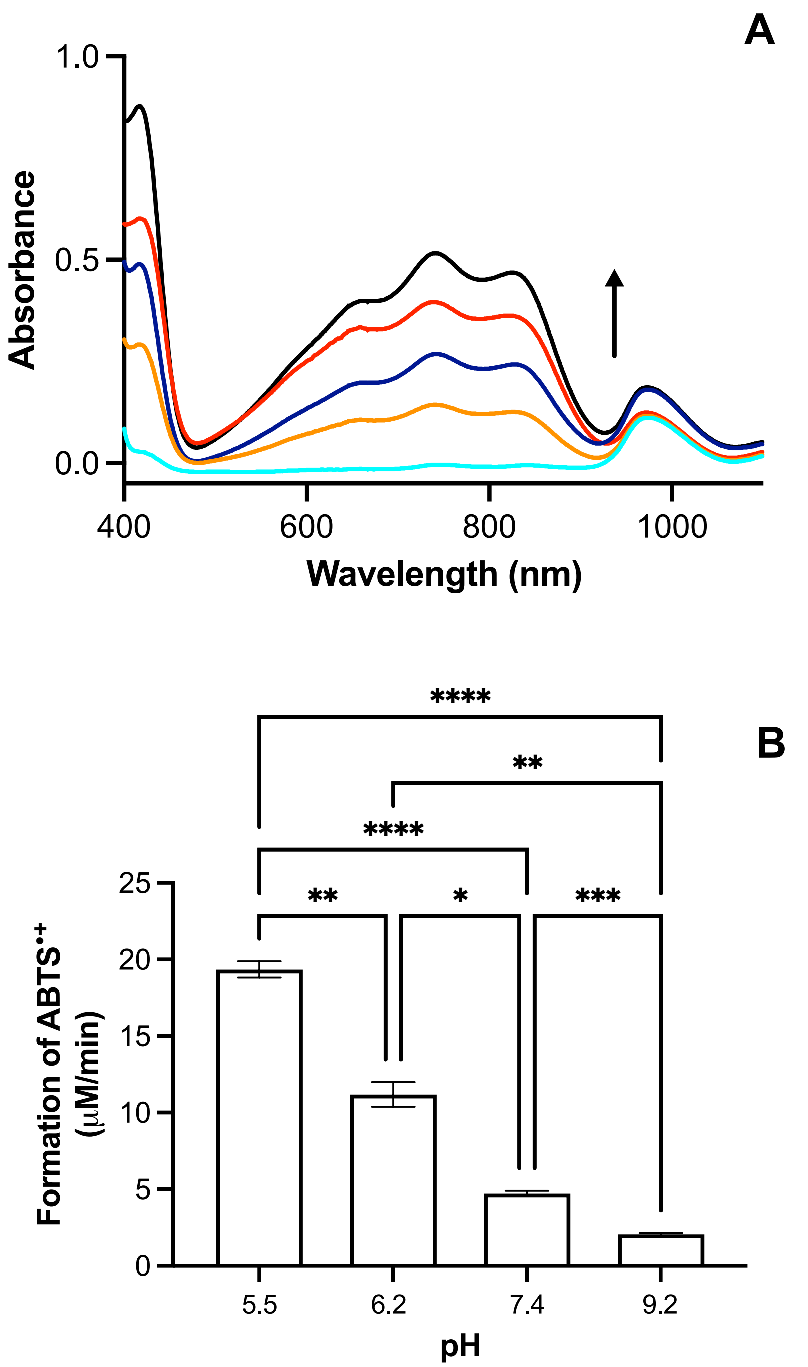

Figure S1. Enzymatic activity of HRP induces one electron oxidation of $ABTS^{2-}$ to its cation radical ($ABTS^{\bullet +}$). Formation of $ABTS^{\bullet +}$ from ABTS by HRP/H_2O_2 was assessed by visible spectrophotometry. Mixtures of 2 nM HRP, 100 μM H_2O_2, and 1 mM ABTS were incubated at 25 ºC for 3 min in 100 mM phosphate (pH 5.5, 6.2, and 7.4) or 100 mM ammonium formate buffer (pH 9.2). Panel A: Visible spectra recorded during the incubation of the HRP/H_2O_2/ABTS system at pH 5.5, with the cyan and black spectra showing data obtained prior to initiation of the reaction and at 3 min, respectively. Panel B: Rate of formation of $ABTS^{\bullet +}$ determined from the initial slope of the absorbance – incubation time plots. In panel B, statistical differences are indicated as follows: * $p < 0.05$, ** $p < 0.01$, *** $p < 0.001$, and **** $p < 0.0001$.

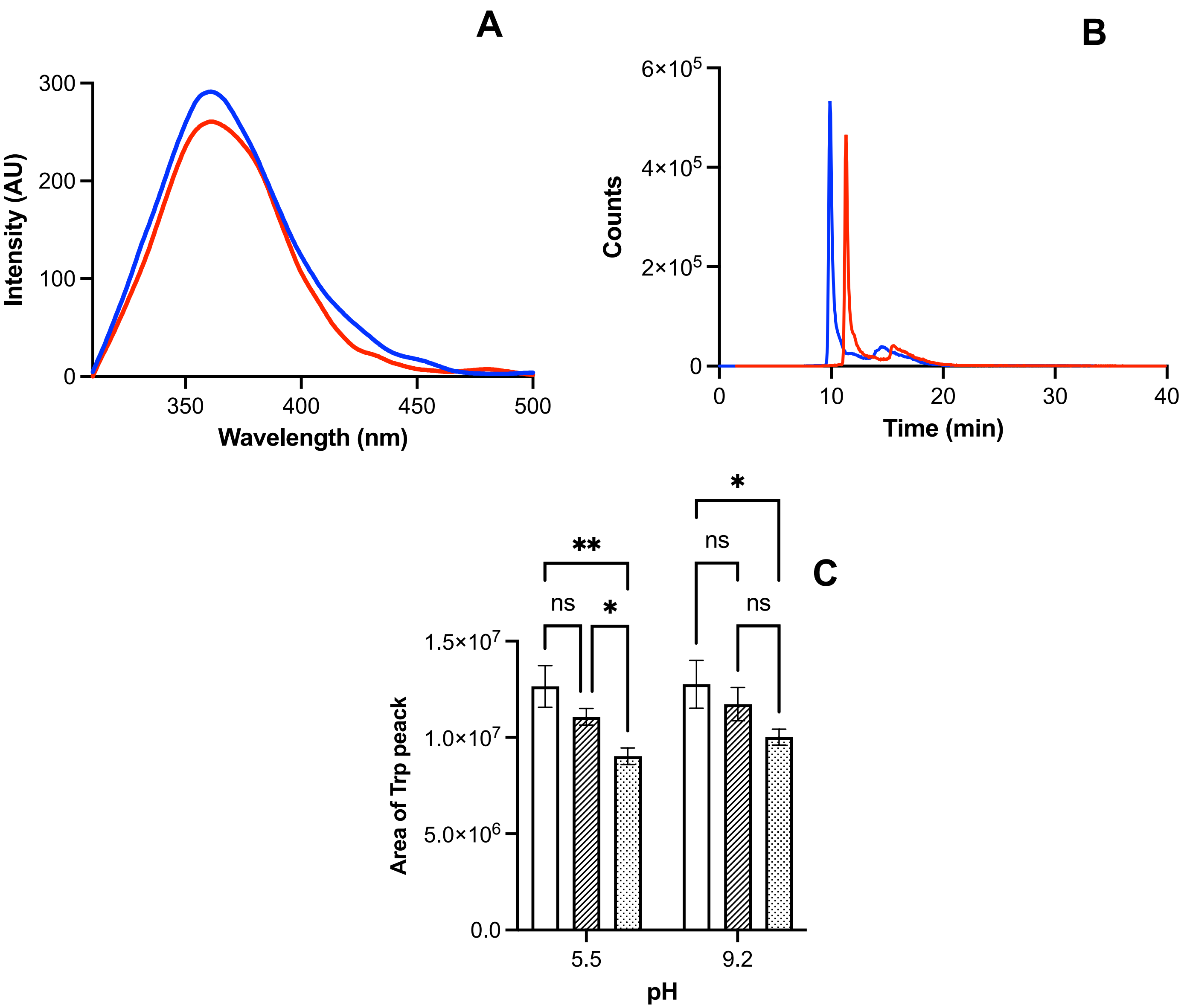

Figure S2. Trp consumption measured by fluorescence and LC-MS/MS. Panel A: Fluorescence spectra of Trp measured in solutions diluted 400-fold before (blue) and after (red) 2 min incubation of 4 mM Trp with 0.65 µM HRP, 500 µM H_2O_2 at pH 5.5. Panel B: Extracted ion chromatogram for *m/z* 205 → 188 transition corresponding to Trp fragmentation, measured in solutions of 4 mM Trp diluted 400-fold before (blue) and after (red) 2 min of reaction with 0.65 µM HRP, 500 µM, at pH 5.5. For clarity, the chromatograms in panel C are presented with an artificial 2 min offset relative to each other. Panel C: Area under the curve of the Trp peak detected by LC-MS/MS from solutions of 4 mM Trp (diluted 400-fold) at 0 min (white), and after 2 min (black lines bars), and 2 h (black dot bars) incubations with 0.65 µM HRP, and 500 µM H_2O_2, at pH 5.5 and 9.2. In panel D, results showing statistical differences with $p < 0.1$ and $p < 0.01$ are indicated with * and **, respectively.

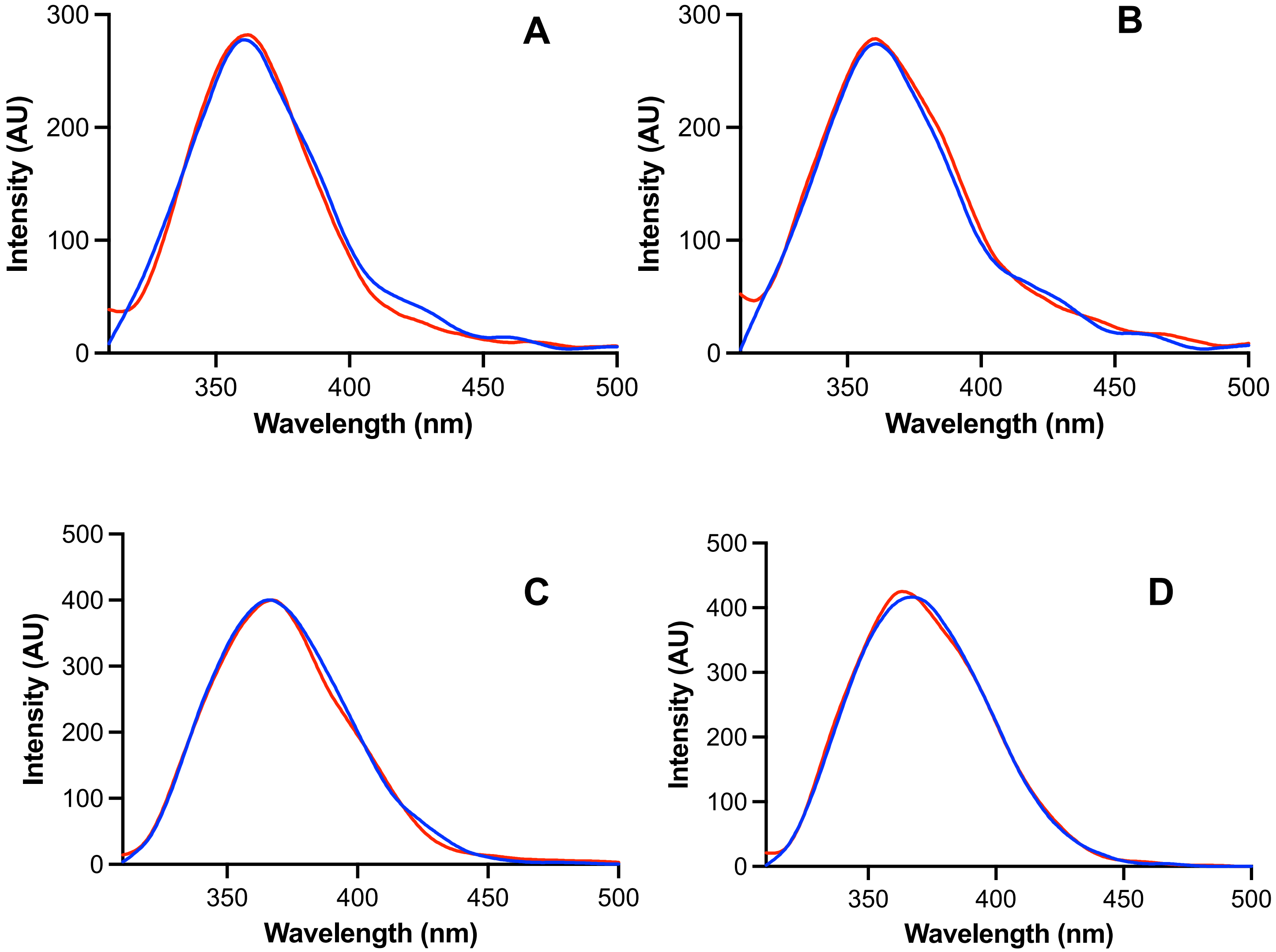

Figure S3. Panel A: FL spectra of a 4 mM Trp solution (diluted 400-fold) registered before (red line) and after 2 min incubations with 500 μM H_2O_2 (blue line) in the absence of HRP, at pH 5.5. Panel B: FL spectra of a 4 mM Trp solution (diluted 400-fold) registered before (red line) and after 2 min incubation with 0.65 μM HRP (blue line) in the absence of H_2O_2, at pH 5.5. Panels C and D show FL results obtained for similar solutions that panels A, and B, but prepared at pH 9.2. FL spectra were registered using 290 nm as the excitation wavelength.

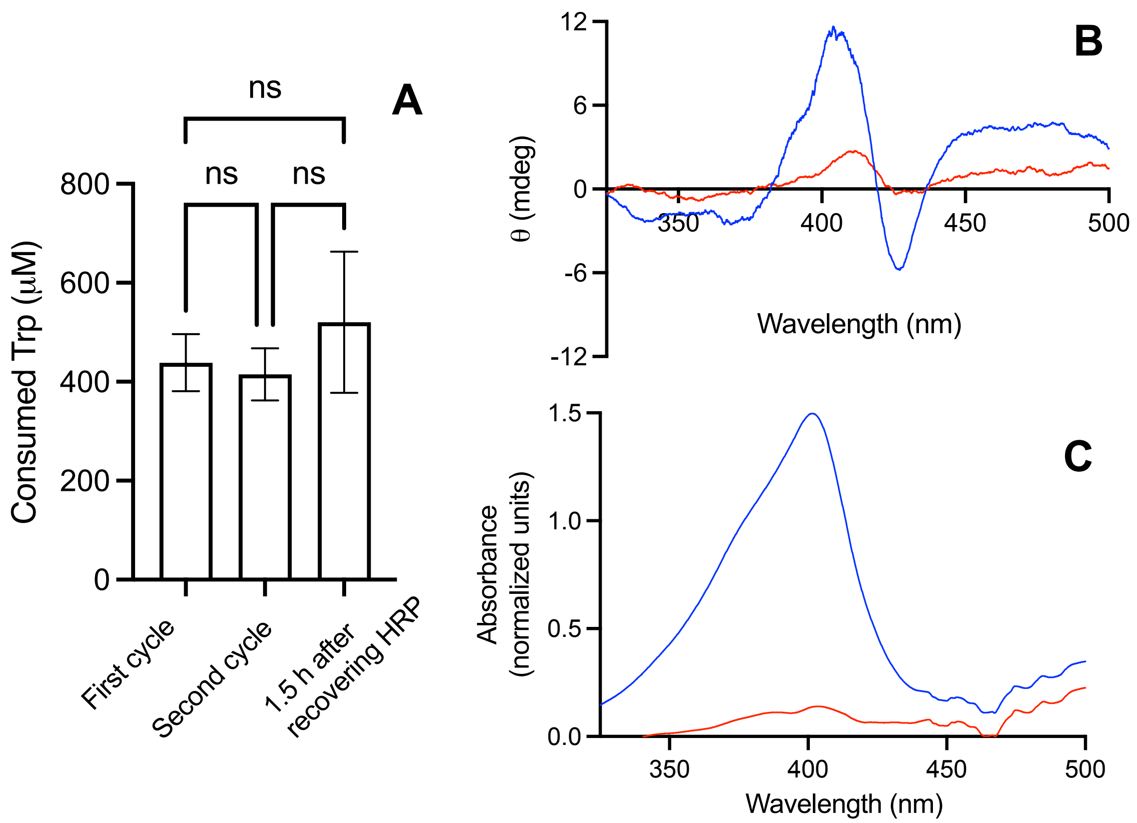

Figure S4. Panel A: Consumption of Trp determined after incubation (2 min) of solutions of 4 mM Trp, 0.65 µM HRP and 500 µM H_2O_2 (pH 9.2) in the first cycle of reaction, and after recovering the enzyme (second cycle). Consumption of Trp was also determined after 1.5 h of HRP recovering. n.s. = data without statistical significance. Panels B, and C show magnetic circular dichroism (MCD), and UV-vis spectra, respectively, obtained using a positive field of 15 kG of a stock solution of HRP (18 µM, blue line), and recovered HRP from incubated Trp/HRP/H_2O_2 solutions for 2 min at pH 9.2 (red line). Concentration of recovered HRP is estimated between 1 and 2 µM.

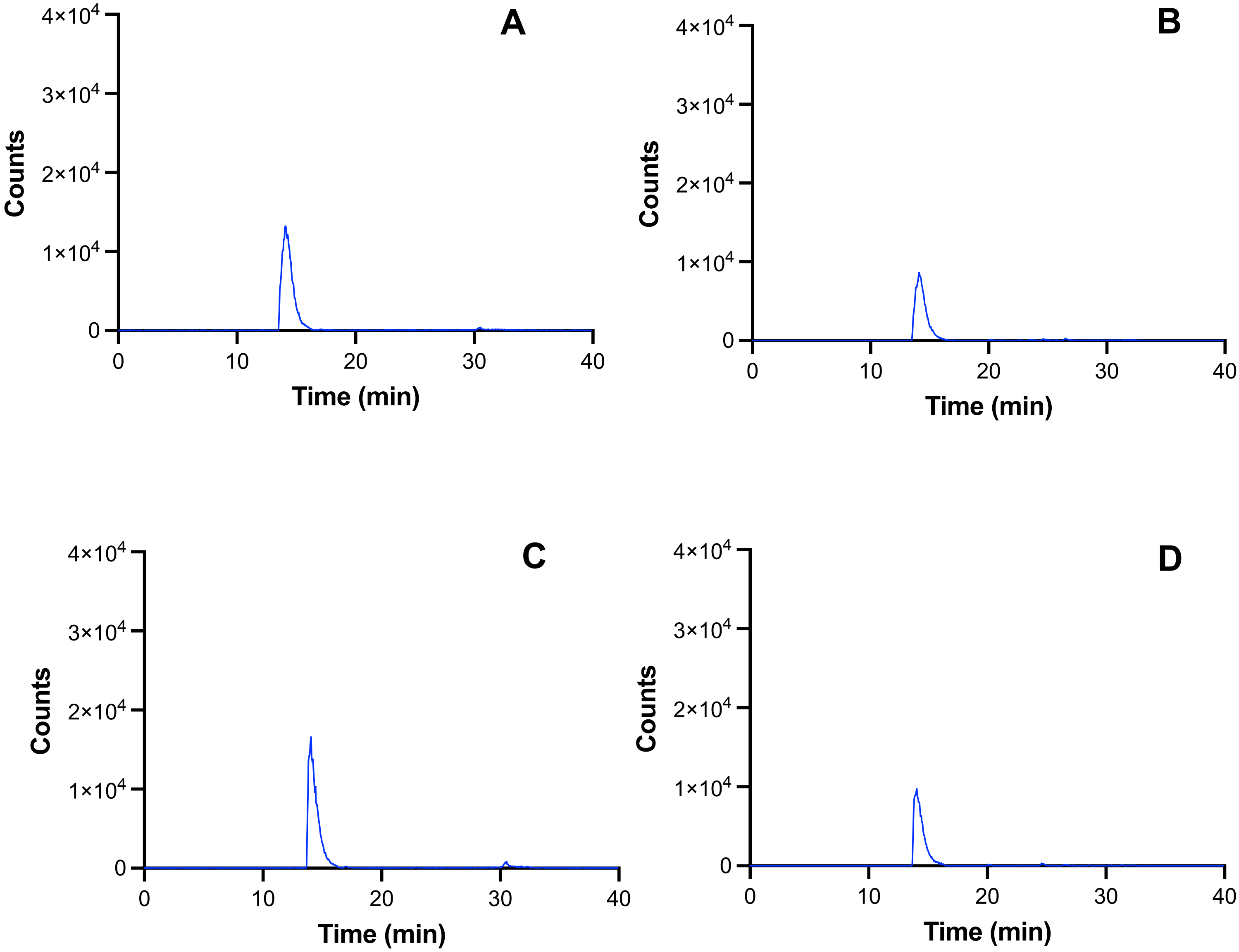

Figure S5. LC-MS/MS analysis for di-Trp signals registered in controls (Trp solutions) at pH 9.2. Panels A and B show the chromatograms registered using the 407 → 203 and 407 → 390 transitions, respectively, for solutions corresponding to incubations of 4 mM Trp for 2 min in the presence of 500 µM H_2O_2 (in the absence of HRP). Panels C and Panel D show chromatograms registered with 407 → 203 and 407 → 390 transitions, respectively, for solutions of 4 mM Trp incubated for 2 min with 0.65 µM HRP (in the absence of H_2O_2).

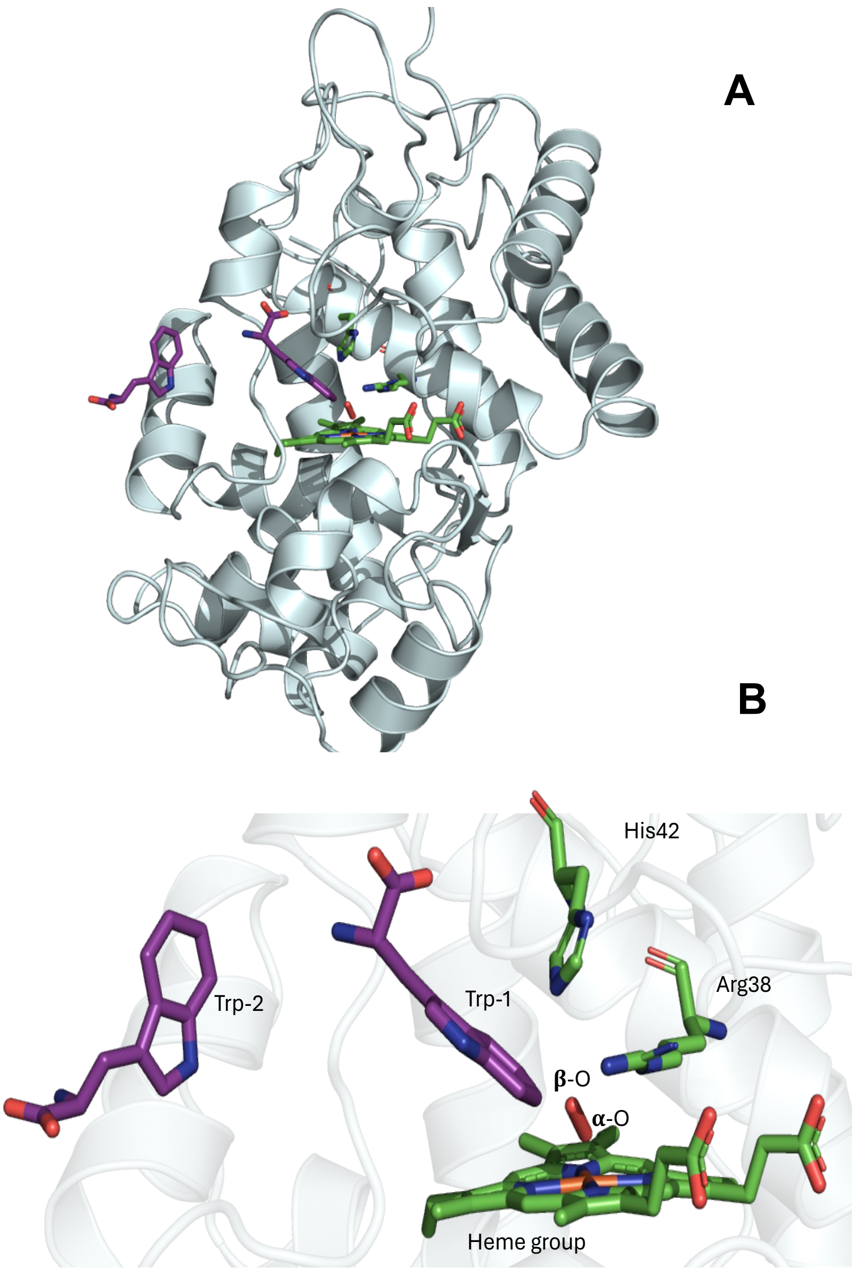

Figure S6. Docking analysis of the Trp/HRP/H_2O_2 complex including a 1:2 stoichiometry (HRP:2Trp). Panels A and B depict the overview of the complex and the zoom of the zone were the two Trp residues (purple) interact with the heme group, His42, and Arg38. The complex was simulated at pH 5.5 using the crystal structure of the enzyme with PDB entry = 1HCH.

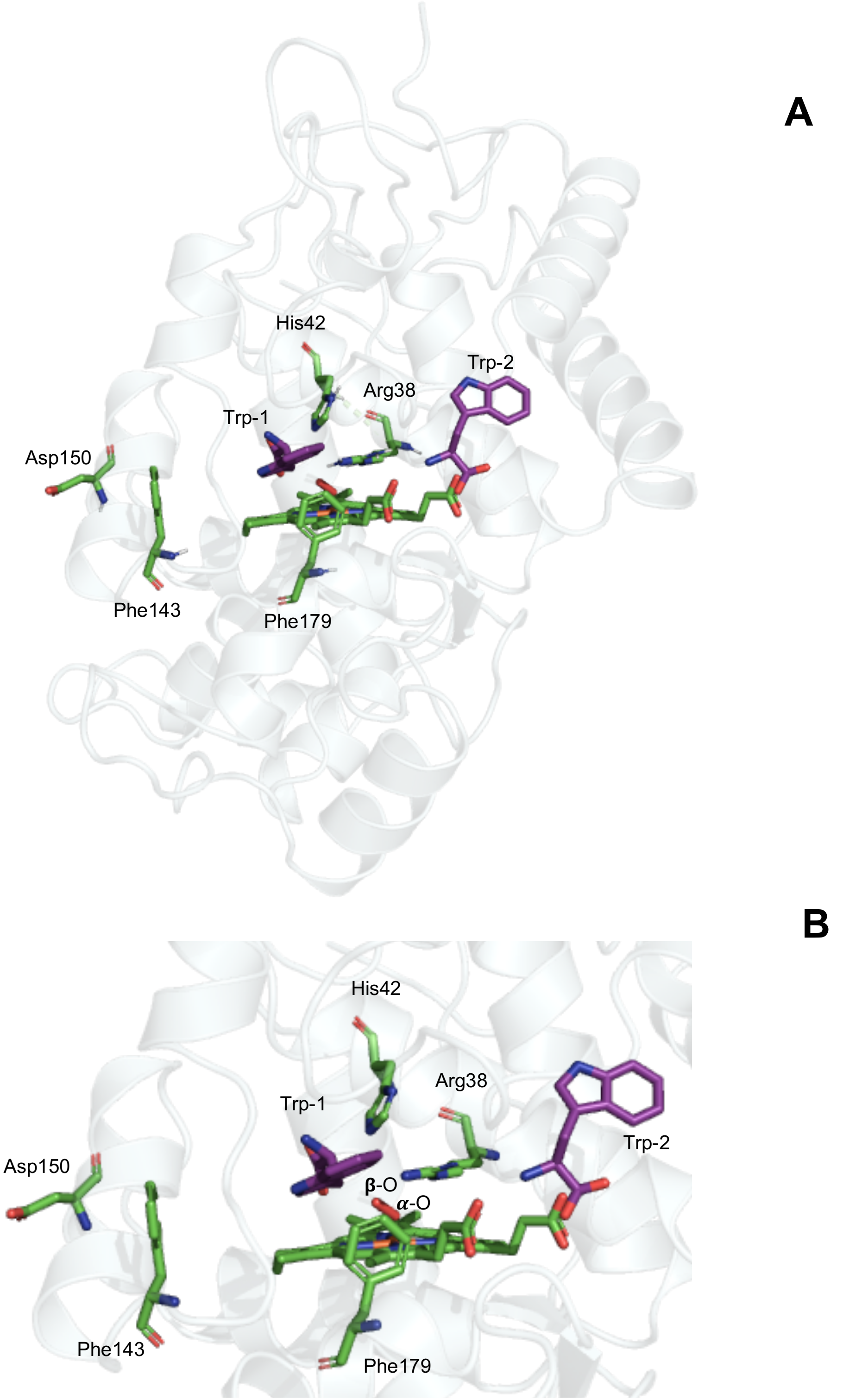

Figure S7. Docking analysis of the Trp/HRP/H_2O_2 complex including a 1:2 stoichiometry (HRP:2Trp), showing the residues of HRP with principal interactions with Trp. Panels A and B depict the overview of the complex and the zoom of the zone were Trp residues interact with the heme group, His42, Arg38, and residues indicated in Table 2. The complex was simulated at pH 9.2 using the crystal structure of the enzyme with PDB entry = 1HCH.
